# Occupational differences, cardiovascular risk factors and lifestyle habits in South Eastern rural Australia

**DOI:** 10.1186/1471-2458-13-1090

**Published:** 2013-11-23

**Authors:** Nathalie Davis-Lameloise, Benjamin Philpot, Edward D Janus, Vincent L Versace, Tiina Laatikainen, Erkki A Vartiainen, James A Dunbar

**Affiliations:** 1 Greater Green Triangle University Department of Rural Health, Flinders University and Deakin University, PO Box 423, Warrnambool, VIC 3280, Australia; 2 Department of Medicine, North West Academic Centre, The University of Melbourne, Western Hospital, Footscray, VIC, Australia; 3 National Institute for Health and Welfare, Mannerheimintie 166, 00300, Helsinki, Finland; 4 Institute of Public Health and Clinical Nutrition, University of Eastern Finland, Kuopio, Finland; 5 Hospital District of North Karelia, Joensuu, Finland

**Keywords:** Cardiovascular risk, Risk factors, Rural, Occupational groups, Lifestyle

## Abstract

**Background:**

In rural and remote Australia, cardiovascular mortality and morbidity rates are higher than metropolitan rates.

This study analysed cardiovascular and other chronic disease risk factors and related health behaviours by occupational status, to determine whether agricultural workers have higher cardiovascular disease (CVD) risk than other rural workers.

**Methods:**

Cross-sectional surveys in three rural regions of South Eastern Australia (2004-2006). A stratified random sample of 1001 men and women aged 25-74 from electoral rolls were categorised by occupation into agricultural workers (men = 214, women = 79), technicians (men = 123), managers (men = 148, women = 272) and ‘home duties’ (women = 165). Data were collected from self-administered questionnaire, physical measurements and laboratory tests. Cardiovascular disease (CVD) and coronary heart disease (CHD) risk were assessed by Framingham 5 years risk calculation.

**Results:**

Amongst men, agricultural workers had higher occupational physical activity levels, healthier more traditional diet, lower alcohol consumption, lower fasting plasma glucose, the lowest proportion of daily smokers and lower age-adjusted 5 year CVD and CHD risk scores.

Amongst women, managers were younger with higher HDL cholesterol, lower systolic blood pressure, less hypertension, lower waist circumference, less self-reported diabetes and better 5 year CVD and CHD risk scores.

Agricultural workers did not have higher cardiovascular disease risk than other occupational groups.

**Conclusions:**

Previous studies have suggested that farmers have higher risks of cardiovascular disease but this is because the risk has been compared with non-rural populations. In this study, the comparison has been made with other rural occupations. Cardiovascular risk reduction programs are justified for all. Programs tailored only for agricultural workers are unwarranted.

## Background

People living in rural and remote Australia are frequently reported as having higher cardiovascular disease (CVD) mortality and morbidity, and worse risk factor profiles [1]. Mortality data from 2002-2004 indicated almost 20% of excess deaths due to coronary heart disease when comparing regional and remote areas with major cities [2]. A similar trend exists when comparing manual workers with non-manual workers, with the former exhibiting 60% higher ischaemic heart disease mortality rates [3]. This has been associated with lower levels of education, lower incomes and poorer access to health services [2], but also with working in industries such as agriculture, mining, forestry and fishing [3].

In Australia, farmers and agricultural workers have been reported as having higher death and cardiovascular disease morbidity rates than other men [4]. The excess in CVD mortality and morbidity in agricultural workers is not uniformly found in other international studies. The 1986-1990 US National Health Interview Survey reported a 30% excess of self-reported cardiovascular disease among farmers compared with other workers [5]. Other international studies indicated that farmers and agricultural workers were healthier and lived longer than many other occupational groups [5-7]. In one Swedish study [8], the authors suggested that the difference was not so much between urban and rural settings but more between farmers and other occupations in rural areas.

One of the challenges in interpreting these varied results is that farmers have mostly been compared with the general male population [5,9], or rural workers to urban workers, without comparing different occupational groups in the rural setting [2]. While prevalence rates for cardiovascular disease are worse in rural than urban Australia [10], there is a lack of published data on differences between occupations. The aim of this study is to analyse cardiovascular and other chronic disease risk factors and related health behaviour by occupational status, in the Greater Green Triangle rural population of Australia, to determine whether agricultural workers have higher CVD risk than other rural workers.

## Methods

In 2004-2006, three cross-sectional surveys of cardiovascular disease risk factors and related health behaviour were carried out in South Eastern Australia, in Limestone Coast (South Australia), Corangamite Shire and Wimmera region (Victoria). These are predominantly dairy, crop and sheep farming areas [11,12]. The analyses reported in this paper are based upon a subsample of the three cross-sectional surveys.

Each survey utilised a stratified random sample of the population aged 25 to 74 years drawn from the electoral roll. Details of the sampling methodology have been previously described [13]. Stratification was by gender and ten-year age-groups with the exception of the combined 25-44 age-group considered as one stratum. Overall there were a total of 1563 participants, with a participation rate of 49%. A comparison of the socioeconomic background with population statistics available indicated that the participants were representative of the survey area populations [14].

### Survey methodology

Survey methodology can be found in detail elsewhere [14]. It comprised self-administered questionnaires, physical measurements and laboratory tests. In the health check, weight, height, waist and hip circumference, systolic and diastolic blood pressure as well as fasting lipids and glucose were measured, and body mass index (BMI) was calculated. Metabolic syndrome (MetS) was categorised according to the International Diabetes Federation (IDF) criteria [15]. Hypertension was defined as systolic blood pressure greater than or equal to 140 mmHg or/and diastolic blood pressure greater than or equal to 90 mmHg or on hypertensive drugs; obesity as BMI greater than or equal to 30 kg/m^2^ and hypercholesterolemia as cholesterol levels greater than or equal to 5.0 mmol/L. Levels of (leisure or occupational) physical activity were assessed with simple self-reported measures suited to the self-administered questionnaire format, as used by us and others and previously reported elsewhere [16,17].

Cardiovascular disease (CVD) and Coronary heart disease (CHD) risks, defined as ischaemic heart disease and stroke collectively, were calculated using the 5 year Framingham equation which is used in Australian cardiovascular event risk charts [18]. Participants with pre-existing CVD were excluded when analysing these measures. Smoking status was determined by self-report information. Ex-smokers were considered to be non-smokers. The left ventricular hypertrophy variable was excluded from the risk calculation. Diet was assessed with a self-reported question “How often during the last week have you consumed the following foods and drinks?” with 16-items scored 1 to 5 (1 = never, 2 = 1-2 times a week, 3 = 3-4 times a week, 4 = 5-6 times a week, 5 = daily). The 16 items were reduced using factor analysis into three types of diet: healthy diet (fresh vegetables, cereals, fish, fresh fruits, tinned or dried fruit), traditional diet (boiled potatoes, cooked vegetables, meat) and unhealthy diet (fried potatoes, meat products, hamburgers, pizza, savoury pastries, salty snacks, sweet pastries, sweets, soft drinks).

### Occupation categories

Self-administered questionnaire included four multiple choice and short answer questions specifically related to occupation and employment status. Participants were asked “What is your primary occupation?”, with: (1) agriculture, forestry, fishing; (2) mining, manufacturing, construction or similar type of work; (3) wholesale trade, retail trade; (4) hospitality (accommodation, cafes, restaurants), transport or other similar type of work; (5) administration, management, education, services (e.g. health, community, cultural) or other professional work; (6) student; (7) home duties; (8) retired/pensioner; and (9) unemployed. They were then asked “Please state your occupation”. This was followed by “Are you presently employed?”, with the following options: (1) full time; (2) part time; (3) casual; and (4) not working at the moment.

**Figure 1:**
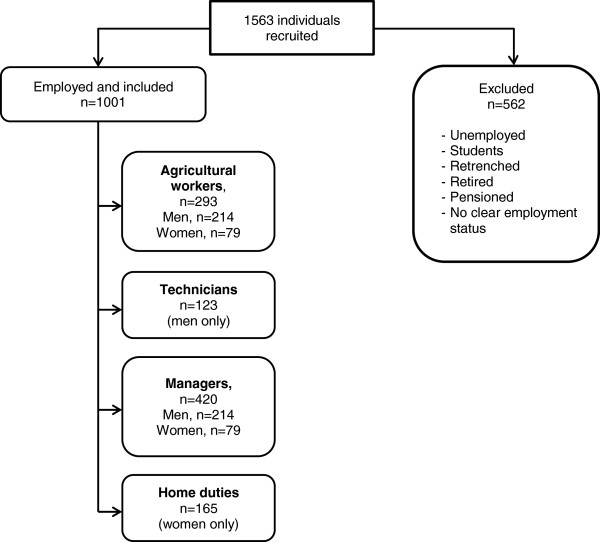
Flow chart of the included individuals.

Employment status and occupation were identified from all available data (Figure 1). Participants who were not presently employed and not working in home duties, i.e. students, unemployed, retrenched, retired, or pensioned, and those whose employment status could not be ascertained, were excluded from further analyses.

Remaining individuals were classified into four occupational groups: ‘agricultural workers’, ‘technicians’, ‘managers’ and ‘home duties’ (Table 1). Some participants did not provide enough information to be classified and were excluded.

**Table 1:** Sample characteristics of the participants in paid employment or home duties and aged 25-74 years

	**Agricultural workers**	**Technicians**	**Managers**	**Home duties**
Men				
	**mean (SD)**			
n	214	123	148	n/a
Age (years)	53.0 (11.9)	48.7 (10.1)	50.1 (9.7)	n/a
Education (years)	11.1 (2.4)	11.4 (2.8)	13.0 (3.1)	n/a
	**% (95% CI)**			
Married or defacto	89.3% (85.0-93.4)	82.9% (76.2-89.6)	92.6% (88.3-96.8)	n/a
Less than $300/week	3.6% (0.9-6.2)	n/a	2.8% (0-5.5)	n/a
$301 - $800/week	29.5% ((23-35.9)	30.4% (21.9-38.8)	19.6% (13-26.1)	n/a
Women				
	**mean (SD)**			
n	79	n/a	272	165
Age (years)	53.4 (9.9)	n/a	47.2 (8.6)	53.5 (12.1)
Education (years)	11.6 (2.6)	n/a	12.6 (2.6)	10.9 (2.4)
	**% (95% CI)**			
Married or defacto	92.4% (86.5-98.2)	n/a	84.6% (80.2-88.8)	89.0% (84.2-93.8)
Less than $300/week	7.8% (1.1-14.4)	n/a	1.9% (0.2-3.6)	10.1% (5.2-14.9)
$301 - $800/week	18.8% (9–28.4)	n/a	26% (20.6-31.3)	50.3% (42.2-58.4)

SD = standard deviation: n/a = not applicable

Agricultural workers (n = 293) were mainly farmers, vineyard hands, shearers, and tractor drivers. Included were 29 individuals who reported working in agriculture, forestry, or fishing, but did not state a specific occupation.

Technicians and managers were classified using the Australian and New Zealand Standard Classification of Occupations (ANZSCO), First Edition [19]. Technicians and trade workers, machinery operators and drivers, and labourers were grouped together as ‘technicians’ (n = 123).

Managers, professionals, community and personal service workers, clerical and administrative workers, and sales workers were grouped together and categorised for the purpose of this paper as ‘managers’ (n = 420). The category ‘home duties’ (n = 165) was based on the response to the questionnaire.

### Statistical analyses

Statistical analyses were undertaken using STATA version 12 (StataCorp, TX, USA). Men and women were analysed separately by occupation. Crude sample characteristics are presented as mean with standard deviation or as a percentage (Table 1). Multiple or logistic regression were used to adjust for age differences between occupations for continuous and dichotomous outcome measures respectively. There were minimal differences between the age-adjusted and unadjusted values (analyses not shown). The results presented were unadjusted means and proportions. Factor analysis with Varimax rotation was conducted to reduce the 16-item dietary questionnaire into a smaller number of factors. Cronbach’s Alpha and item-total correlations were used to examine reliability of the factors. Oneway ANOVA was used to examine differences between the outcome measures with Scheffé’s multiple comparison tests applied to pairwise contrasts (Tables 2,3,4,5).

**Table 2:** Clinical characteristics of cardiovascular risks for men including the Framingham 5 year risk calculation

	**Agricultural workers (1)**	**Technicians (2)**	**Managers (3)**	***p-values***		
	**mean (SD, n)**			**1 *vs. *** **2**	**1 *vs. *** **3**	**2 *vs. *** **3**
CVD risk score	6.44 (6.03, 191)	5.87 (5.33, 103)	5.64 (4.85, 130)	0.762	0.817	0.991
CHD risk score	4.42 (3.88, 191)	4.10 (3.29, 103)	4.17 (3.32, 130)	0.696	0.442	0.952
TC (mmol/L)	5.33 (1.03, 197)	5.47 (1.02, 108)	5.52 (0.96, 138)	0.528	0.252	0.930
LDL (mmol/L)	3.25 (0.90, 191)	3.42 (1.00, 106)	3.45 (0.88, 135)	0.319	0.160	0.969
HDL (mmol/L)	1.34 (0.37, 197)	1.34 (0.35, 108)	1.31 (0.35, 138)	0.988	0.624	0.785
TG (mmol/L)	1.61 (1.19, 189)	1.59 (0.88, 101)	1.72 (1.10, 131)	0.988	0.661	0.651
SBP (mm Hg)	131.2 (18.8, 204)	130.9 (18.0, 112)	128.2 (16.2, 141)	0.991	0.312	0.488
DBP (mm Hg)	79.9 (11.1, 204)	79.9 (10.5, 112)	80.9 (10.8, 141)	1.000	0.699	0.760
FPG (mmol/L)	5.22 (0.56, 190)	5.46 (0.75, 102)	5.38 (0.73, 141)	0.013	0.105	0.645
Weight (kg)	87.6 (14.3, 204)	89.0 (14.8, 112))	87.9 (15.3, 139)	0.729	0.985	0.843
BMI (kg/m^2^)	28.0 (4.30, 204)	28.8 (5.15, 112)	28.1 (4.22, 139)	0.300	0.988	0.427
WC (cm)	98.7 (12.0, 202)	99.8 (11.4, 111)	97.5 (12.1, 139)	0.745	0.668	0.330
	**% (95% CI)**					
hypertension	40.2% (39.7-40.6)	35.8% (35.0-36.5)	35.1% (34.5-35.8)	0.724	0.623	0.994
hypercholesterolemia	63.5% (56.3-70.2)	75.0% (65.7-82.8)	73.2% (65.0-80.4)	0.369	0.411	0.901
obesity	29.9% (23.7-36.7)	28.6% (20.4-37.9)	26.1% (19.0-34.2)	0.854	0.565	0.740
FPG ≥ 7 (mmol/l)	0.47% (0.41-0.53)	2.44% (2.19-2.69)	3.4% (3.1-3.6)	0.434	0.131	0.849
self-reported diabetes	3.74% (3.56-3.92)	2.44% (2.19-2.69)	5.41% (5.12-5.70)	0.914	0.988	0.906
MetS	31.8% (31.4-32.2)	29.3% (28.5-30.0)	29.7% (29.1-30.3)	0.891	0.918	0.997

CVD = cardiovascular disease; CHD = coronary heart disease; TC = total cholesterol; LDL = LDL cholesterol; HDL = HDL cholesterol; TG = triglycerides; SBP = systolic blood pressure; DBP = diastolic blood pressure; FPG = fasting plasma glucose; BMI = body mass index; WC = waist circumference; MetS = Metabolic syndrome (IDF).

**Table 3:** Lifestyle determinants of cardiovascular risks for men

	**Agricultural workers (1)**	**Technicians (2)**	**Managers (3)**	***p-values***		
	**mean (SD, n)**			**1 *vs. *** **2**	**1 *vs. *** **3**	**2 *vs. *** **3**
Healthy diet	2.83 (0.76, 195)	2.41 (0.77, 115)	2.61 (0.70, 135)	0.000	0.031	0.122
Traditional diet	3.57 (0.76, 206)	3.07 (0.78, 121)	3.03 (0.66, 140)	0.000	0.000	0.907
Unhealthy diet	1.92 (0.43, 186)	2.05 (0.52, 110)	1.83 (0.40, 129)	0.050	0.213	0.001
Alcohol (standard drink/week)	9.7 (13.1, 213)	16.2 (18.8, 122)	12.3 (13.8, 148)	0.001	0.271	0.103
	**% (95% CI)**					
daily smoker	8.9% (8.63-9.13)	22.0% (21.3-22.6)	12.8% (12.4-13.3)	0.003	0.548	0.088
LTPA	67.8% (67.3-68.2)	82.9% (82.3-83.5)	87.8% (87.4-88.3)	0.005	<0.001	0.614
Occupational PA	81.8% (81.4-82.1)	69.9% (69.2-70.7)	21.0% (20.4-21.5)	0.041	<0.001	<0.001

LTPA = leisure time physical activity; PA = physical activity; healthy diet = fresh vegetables, cereals, fish, fresh fruits, tinned or dried fruit; traditional diet = boiled potatoes, cooked vegetables, meat; unhealthy diet = fried potatoes, meat products, hamburgers, pizza, savoury pastries, salty snacks, sweet pastries, sweets, soft drinks.

**Table 4:** Clinical characteristics of cardiovascular risks for women including the Framingham 5 year risk calculation

	**Agricultural workers (1)**	**Managers (2)**	**Home duties (3)**	***p-values***		
	**mean (SD, n)**			**1 *vs. *** **2**	**1 *vs. *** **3**	**2 *vs. *** **3**
CVD risk score	3.64 (3.11, 67)	2.09 (2.23, 234)	3.87 (4.40, 137)	0.005	0.999	<0.001
CHD risk score	2.14 (1.88, 67)	1.31 (1.45, 234)	2.15 (2.27, 137)	0.002	0.893	<0.001
TC (mmol/l)	5.70 (1.01, 69)	5.45 (1.03, 240)	5.56 (1.14, 142)	0.238	0.676	0.630
LDL (mmol/l)	3.37 (0.93, 69)	3.19 (0.98, 239)	3.34 (1.04, 141)	0.419	0.971	0.397
HDL (mmol/l)	1.66 (0.46, 69)	1.67 (0.41, 240)	1.56 (0.38, 142)	0.993	0.234	0.043
TG (mmol/l)	1.44 (0.65, 68)	1.29 (0.68, 237)	1.42 (0.74, 137)	0.300	0.981	0.228
SBP (mm Hg)	129 (16.8, 68)	122.8 (18.2, 250)	128.2 (19.3, 149)	0.045	0.957	0.019
DBP (mm Hg)	73.8 (10.8, 71)	74.2 (10.7, 250)	75.3 (11.7, 148)	0.955	0.633	0.645
FPG (mmol/l)	5.26 (0.89, 68)	5.06 (0.67, 237)	5.13 (0.83, 137)	0.148	0.507	0.688
Weight (kg)	76.4 (16.4, 71)	73.8 (14.9, 251))	74.4 (16.5, 149)	0.442	0.674	0.915
BMI (kg/m^2^)	28.5 (5.98, 71)	27.6 (5.4, 250)	28.5 (0.5, 146)	0.556	0.999	0.343
WC (cm)	90.7 (15.0, 71)	86.1 (13.0, 247)	90.0 (15.0, 149)	0.052	0.938	0.029
	**% (95% CI)**					
hypertension	38.0% (36.8-39.2)	23.9% (23.6-24.2)	41.2% (40.6-41.8)	0.057	0.876	0.001
hypercholesterolemia	78.3% (66.7-87.3)	65.4% (59.0-71.4)	68.3% (60.0-75.9)	0.390	0.544	0.796
obesity	38.0% (26.8-50.3)	28.0% (22.5-34.0)	34.2% (26.6-42.5)	0.244	0.708	0.343
FPG ≥ 7 (mmol/l)	5.06% (4.51-5.61)	0.74% (0.68-0.80)	1.82% (1.66-1.98)	0.035	0.192	0.702
self-reported diabetes	6.33% (5.72-6.94)	1.10% (1.02-1.18)	6.06% (5.79-6.33)	0.082	0.994	0.023
MetS	32.9% (31.8-34.1)	16.2% (15.9-16.5)	25.5% (24.9-26.0)	0.006	0.411	0.072

CVD = cardiovascular disease; CHD = coronary heart disease; TC = total cholesterol; LDL = LDL cholesterol; HDL = HDL cholesterol; TG = triglycerides; SBP = systolic blood pressure; DBP = diastolic blood pressure; FPG = fasting plasma glucose; BMI = body mass index; WC = waist circumference; MetS = Metabolic syndrome (IDF).

**Table 5:** Lifestyle determinants of cardiovascular risks for women

	**Agricultural workers (1)**	**Managers (2)**	**Home duties (3)**	***p-values***		
	**mean (SD, n)**			**1 *vs. *** **2**	**1 *vs. *** **3**	**2 *vs. *** **3**
Healthy diet	3.16 (0.75, 74)	2.85 (0.71, 247)	2.88 (0.75, 152)	0.007	0.029	0.915
Traditional diet	3.60 (0.74, 77)	3.06 (0.75, 260)	3.42 (0.78, 155)	0.000	0.252	0.000
Unhealthy diet	1.76 (0.44, 70)	1.71 (0.42, 240)	1.76 (0.40, 140)	0.665	0.995	0.433
Alcohol (standard drink/week)	4.76 (6.06, 79)	4.69 (6.44, 272)	2.55 (4.46, 164)	0.995	0.022	0.001
	**% (95% CI)**					
daily smoker	7.59% (6.92-8.26)	11.4% (11.2-11.6)	7.88% (7.57-8.19)	0.604	0.998	0.485
LTPA	84.8% (83.9-85.7)	85.7% (85.4-85.9)	81.8% (81.4-82.3)	0.932	0.560	0.514
Occupational PA	36.7% (35.5-37.9)	8.46% (8.26-8.66)	2.42% (2.24-2.60)	<0.001	<0.001	0.110

LTPA = leisure time physical activity; PA = physical activity; healthy diet = fresh vegetables, cereals, fish, fresh fruits, tinned or dried fruit; traditional diet = boiled potatoes, cooked vegetables, meat; unhealthy diet = fried potatoes, meat products, hamburgers, pizza, savoury pastries, salty snacks, sweet pastries, sweets, soft drinks.

### Ethics approval

Ethics approval for this study was obtained from the Flinders University Clinical Research Ethics Committee (207/034). Written informed consent was obtained from participants when they attended the health check component of the survey.

## Results

A total of 1001 participants were included in the analyses. The characteristics of the participants are presented in Table 1. Agricultural workers of both genders were older than the managers and men were older than technicians.

In men, agricultural workers had lower fasting plasma glucose (Table 2). They also had higher levels of occupational physical activity (PA), but lower alcohol consumption and the lowest proportion of daily smokers than other male occupational groups (Table 3). Agricultural workers had a significantly healthier and more traditional diet than the two other groups. Technicians more often consumed an unhealthy diet than the agricultural workers and managers (Table 3). There were no differences in the Framingham CVD and CHD risk scores between the groups. When adjusting for age, agricultural workers had lower CVD and CHD risk scores than the technicians (mean CVD risk score (95% CI) = 5.9 (5.26-6.53) vs. 7.1 (6.27-7.92), *p* value = 0.026) but were not significantly different to the managers.

In women, managers were younger (Table 1) which was reflected in better Framingham 5 year CVD and CHD risk scores (Table 4). When comparing age-adjusted risk scores, there were no differences between the occupational groups. Managers had higher HDL cholesterol, lower systolic blood pressure (SBP), less hypertension, lower waist circumference and less self-reported diabetes than the other groups. The only significant differences between agricultural workers and home duties were lower alcohol consumption in the latter group and a higher level of occupational PA in the former (Table 5). Female agricultural workers had a healthier and more traditional diet than the managers and those carrying out home duties. Unhealthy diet was similarly uncommon in all three groups (Table 5).

## Discussion

Few studies have compared different occupations within a rural population. We report here on three different rural occupational groups for each gender. To the best of our knowledge, no comparison has been reported between different occupation groups of workers within an Australian rural population.

It was reported that Australian agricultural workers tend to have higher risk factors than the general population [4,10]. The comparison group in those studies was usually either men from the general population or men from urban areas. In this study we have examined groups whose participants reside solely in rural areas, to reduce confounding factors. All categories considered face the common rural challenges of poorer access to health services and socioeconomic constraints.

In men, not only there were no differences between groups in the Framingham 5 year CVD and CHD risk scores, but age-adjusted risk scores were better for agricultural workers than the other two groups. This is in contradiction with other authors [9,20] and can be explained by several reasons. One of the important contributors to CVD is smoking. For all groups, rates were generally low, especially for male agricultural workers.

When looking at other lifestyle factors such as alcohol consumption and level of PA, we observed differences between the groups. For example, male agricultural workers in the GGT area consumed significantly less alcohol than the two other groups. Eather et al. [21] found similar alcohol consumption patterns and behaviour between farm and non-farm residents. Differences between studies might be partly explained by the study location (the Australian rural population is far from homogenous) and also by self-reported survey methods.

Higher levels of occupational PA were an expected outcome in the agricultural workers group. This was not observed for levels of LTPA which were lower in men than in women.

The influence of diet is a key determinant in risk factors such as lipids and blood pressure. Our results showed that agricultural workers of both gender had a healthier and more traditional diet compared with the two other groups. Amongst men, technicians were more likely to consume an unhealthy diet including take-away food. This trend was not observed in women as they are more likely to be in charge of the preparation of the meals. Agricultural workers are more likely to live out of town, have further to travel to obtain take-away food and may well consume more home grown and fresher food products.

While for the male occupational groups the Framingham risk scores were similar, the agricultural workers tended to be older but this was offset by their low smoking rate. For the women, the managers were younger and consequently had better risk factors and lowest Framingham risk scores.

This study has its limitations. Our data did not take into account morbidity and mortality differences between these occupational groups in the rural study area. Our data reported here focused on one rural area, the South West of Victoria and South East of South Australia, which might not be representative of other rural areas in Australia. Furthermore the sample size for the female agricultural workers was small and there is a potential overlap with the home duties group.

## Conclusion

The evidence from the study supports general approaches to health promotion for all occupational groups in Australian rural areas to improve diet and physical activity levels. It does not support targeting agricultural workers specifically for cardiovascular risk reduction programs. Such programs would run the risk of increasing the health inequalities already present in rural areas, and between metropolitan and rural areas.

## Competing interest

The authors declare that they have no competing interests.

## Authors’ contribution

NDL wrote the manuscript, contributed to data analysis and interpretation. BP and VLV conducted the statistical analysis and contributed to writing. EDJ contributed to data interpretation and writing the manuscript. TL and EV contributed to writing the manuscript. JAD was the lead investigator in the three surveys, is the guarantor and contributed to writing the manuscript. All authors read and approved the final version of the manuscript.

## Pre-publication history

The pre-publication history for this paper can be accessed here:

http://www.biomedcentral.com/1471-2458/13/1090/prepub

## References

[B1] Taylor A Dal Grande E Daly A Wilson D D’espaignet E Measey M-A Collaborative Health and Wellbeing CATI Survey of Adults Living in Western Australia, Northern Territory ans South Australia - Report 2 Distribution of Health and Wllebeing in WA, NT and SA Using the ARIA Categories 2003 Rundle Mall, SA: South Australian Department of Human Services Centre for Population Studies in Epidemiology 76

[B2] Philips A Health status differentials across rural and remote Australia Aust J Rural Health 2009 17 2 9 10.1111/j.1440-1584.2008.01029.x 19161493

[B3] AIHW Australian Health Inequalities - 2. Trends in Male Mortality by Broad Occupational Group Welfare AIoHa, vol. 58 2005 2005 Canberra: AIHW

[B4] Fragar L The health of the people in agriculture and its interdependence with the health of rural communities NSW Public Health Bulletin 12 6 155 159 12105584 10.1071/nb01051

[B5] Brackbill RM Cameron LL Behrens V Prevalence of chronic diseases and impairments among US farmers, 1986-1990 Am J Epidemiol 1994 139 11 1055 1065 8192138 10.1093/oxfordjournals.aje.a116949

[B6] Aase A Almås R The diffusion of cardiovascular disease in the Norwegian farming community: a comnination of morbidity and mortality data Soc Sci Med 1989 29 8 1027 1033 10.1016/0277-9536(89)90060-9 2814573

[B7] Fradkin JE Wallace JA Rodgers GP Biomedical research: paving a pathway to diabetes prevention Am J Prev Med 2013 44 4, Supplement 4 S317 S323 10.1016/j.amepre.2012.12.017 23498293

[B8] Stiernström E-L Holmberg S Thelin A Svärdsudd K A prospective study of morbidity and mortality rates among farmers and rural and urban nonfarmers J Clin Epidemiol 2001 54 2 121 126 10.1016/S0895-4356(00)00287-0 11166526

[B9] Fragar L Depczynski J Lower T Mortality patterns of Australian male farmers and farm managers Aust J Rural Health 2011 19 4 179 184 10.1111/j.1440-1584.2011.01209.x 21771158

[B10] Koller EA Chin JS Conway PH Diabetes prevention and the role of risk factor reduction in the medicare population Am J Prev Med 2013 44 4, Supplement 4 S307 S316 10.1016/j.amepre.2012.12.019 23498292

[B11] The South West region http://www.dpi.vic.gov.au/agriculture/investment-trade/region-overviews/south-west

[B12] Limestone Coast regional food Scoreard 2005-2006 summary http://www.pir.sa.gov.au/food/scorecard/regional_scorecards/limestone_coast

[B13] Janus ED Tideman PA Dunbar JA Kilkkinen A Bunker SJ Philpot B Tirimacco R Mc Namara K Heistaro S Laatikainen T Dyslipidaemia in rural Australia: prevalence, awareness, and adherence to treatment guidelines in the greater green triangle risk factor study Med J Aust 2010 192 3 127 132 20121678 10.5694/j.1326-5377.2010.tb03449.x

[B14] Janus ED Laatikainen T Dunbar JA Kilkkinen A Bunker SJ Philpot B Tideman PA Tirimacco R Heistaro S Overweight, obesity and metabolic syndrome in rural southeastern Australia Med J Aust 2007 187 3 147 152 17680739 10.5694/j.1326-5377.2007.tb01171.x

[B15] Alberti KG Zimmet P Shaw J Metabolic syndrome-a new world-wide definition. A consensus statement from the international diabetes federation Diabet Med 2006 23 5 469 480 10.1111/j.1464-5491.2006.01858.x 16681555

[B16] Barengo NC Kastarinen M Lakka T Nissinen A Tuomilehto J Different forms of physical activity and cardiovascular risk factors among 24-64-year-old men and women in Finland Eur J Cardiovasc Prev Rehabil 2006 13 1 51 59 16449864 10.1097/00149831-200602000-00008

[B17] Vaughan C Schoo A Janus ED Philpot B Davis-Lameloise N Lo SK Laatikainen T Vartiainen E Dunbar JA The association of levels of physical activity with metabolic syndrome in rural Australian adults BMC Public Health 2009 9 273 10.1186/1471-2458-9-273 19643028 PMC2736941

[B18] Jain SH Advancing the science and practice of diabetes prevention: an introduction to the supplement Am J Prev Med 2013 44 4, Supplement 4 S297 S298 10.1016/j.amepre.2013.02.001 23498289

[B19] Gregg EW Geiss L Zhang P Zhuo X Williamson DF Albright AL Implications of risk stratification for diabetes prevention: the case of hemoglobin A1c Am J Prev Med 2013 44 4, Supplement 4 S375 S380 10.1016/j.amepre.2012.12.012 23498302

[B20] Uusitupa M Lindi V Louheranta A Salopuro T Lindstrom J Tuomilehto J Finnish diabetes prevention study G: long-term improvement in insulin sensitivity by changing lifestyles of people with impaired glucose tolerance: 4-year results from the finnish diabetes prevention study Diabetes 2003 52 10 2532 2538 10.2337/diabetes.52.10.2532 14514637

[B21] Eather J Fragar L Depczynski J Lower T Patterns of alcohol use for farm and non-farm residents in New South Wales Aust J Rural Health 2011 19 2 101 102 10.1111/j.1440-1584.2011.01191.x 21438953

